# The transcriptome of *Cryptosporidium* oocysts and intracellular stages

**DOI:** 10.1038/s41598-019-44289-x

**Published:** 2019-05-27

**Authors:** Lucas V. S. Matos, John McEvoy, Saul Tzipori, Katia D. S. Bresciani, Giovanni Widmer

**Affiliations:** 1 Department of Infectious Disease & Global Health, Cummings School of Veterinary Medicine at Tufts University, North Grafton, Massachusetts, 01536 USA; 2 Universidade Estadual Paulista (UNESP), Faculdade de Ciências Agrárias e Veterinárias, Jaboticabal, São Paulo, Brazil; 3 Department of Microbiological Sciences, North Dakota State University, Fargo, USA; 4 Universidade Estadual Paulista (UNESP), Faculdade de Medicina Veterinária, Araçatuba, São Paulo, Brazil

**Keywords:** Pathogens, Molecular biology

## Abstract

Human cryptosporidiosis is caused primarily by two species of apicomplexan parasites, *Cryptosporidium parvum* and *C. hominis*. Although infection of cell monolayers with sporozoites does not support the complete parasite life cycle, the *in vitro* system is used to study the asexual phase of multiplication, which consists of two generations of merogony. To better understand host-parasite interaction and to gain insight into gene regulatory processes driving the complex life cycle of *Cryptosporidium* parasites, we analyzed the transcriptome of *C. parvum* in oocysts, sporozoites and infected cell monolayers 2–48 h post-infection. Analysis of RNA-Seq data from replicate oocyst, sporozoite and intracellular samples revealed significant differences between transcriptomes expressed outside and inside the host cell. Compared to the transcriptome found in the host cell, the oocyst transcriptome is less diverse. Biological processes significantly over-represented intracellularly relate to biosynthetic processes. Genes significantly overexpressed in oocysts show evidence of specialized functions not found in other Apicomplexa. A more comprehensive view of gene regulation during the *Cryptosporidium* life cycle will require the analysis of later time points during the infection, particularly of the poorly studied sexual phase of the life cycle.

## Introduction

Cryptosporidiosis is recognized as one of the most common enteric infections in infants in sub-Saharan Africa and southeast Asia^1^. The rapid asexual multiplication of the parasite in the intestinal epithelium compromises intestinal function, which can lead to severe diarrhea and have long-term consequences^2^. There are no effective drugs to control cryptosporidiosis. Drug screening is made difficult by the lack of robust culture methods supporting the entire life cycle.

Commonly used methods to culture *Cryptosporidium* parasites^3^ do not support completion of the life cycle, possibly due to inefficient differentiation of gametes and/or deficient fertilization. Oocysts excreted in the feces of naturally or experimentally infected animals can be used to infect cultured epithelial cells. The oocysts release sporozoites which are capable of invading host cells in the intestinal epithelium or in culture. Following invasion, sporozoites transform into trophozoites and divide asexually to generate first-and second-generation meronts in a process known as merogony. Later stages, specifically the sexual phase of the life cycle, do not appear to differentiate consistently in conventional cell monolayers. Progress towards a more stable culture in biphasic and three-dimensional culture systems has been reported^4–7^.

Compared to better studied Apicomplexa, gene regulation during the *Cryptosporidium* life cycle has rarely been studied^8,9^. Reflecting the many technical obstacles to research on these parasites, published *Cryptosporidium* transcriptome analyses based on RNA-Seq are limited to one study of *C. parvum* extra- and intracellular gene expression in calf intestinal epithelium and in culture^9^ and an analysis of the *C. parvum* life cycle transcriptome in organoids grown from small intestine and lung epithelial cells^7^. With the goal of improving our understanding of gene regulation in *Cryptosporidium* parasites, we undertook an RNA-Seq analysis of the *Cryptosporidium* transcriptome at the oocyst, excysted sporozoite, and intracellular stages. To ensure that the transcriptome features identified in our analysis are of general validity, as opposed to being restricted to a particular cell line, parasite isolate, or experimental condition, we included sequence data originating from infections of different cell lines and different *C. parvum* isolates. In addition, sporozoite RNA-Seq data generated by Lippuner *et al*.^9^ were downloaded from the National Center for Biotechnology Information’s Sequence Read Archive and combined with sequence data generated as part of the present study. We identified functional categories overrepresented in the oocyst transcriptome and found these to be indicative of the specialized functions, like long-term survival and delivery of sporozoites into specific GI tract organs. In contrast, transcriptomic data of intracellular parasite stages indicate that the intracellular transcriptome is tailored for transcription and translation, consistent with rapid asexual multiplication during the initial phase of the infection.

To examine whether sporozoite gene expression responds to environmental stimuli, transcriptomic data were acquired from sporozoites incubated under different conditions. Compared to the difference between intra- and extracellular *C. parvum* transcriptome, differences in gene expression between oocysts and sporozoites were relatively small.

## Methods

### Parasites

Figure S1 shows a flowchart of the experimental procedures. Oocyst from three *C. parvum* isolates were used. First, fecal samples from diarrheic newborn calves raised on a farm in Woodstock, Connecticut were screened for the presence of *Cryptosporidium* oocysts and one sample with a high number of oocysts (3 × 10^7^ oocysts/ml) was selected. Oocysts from this unnamed isolate were used to infect four replicate monolayers of IPEC-J2 cell monolayers as described^10^. These monolayers are designated 1M-4M (Table 1). Second, oocysts from isolate TU114^11^ were used to infect four replicate monolayers of MDBK cells (ATCC CCL-22). TU114 originates from a human infection in Uganda. It has been maintained by serial propagation in immunosuppressed mice since 2003. Third, oocysts of *C. parvum* isolate MD^12^ were used to infect a second set of MDBK cell monolayers. MD was originally isolated from a deer and has been maintained by serial propagation in immunosuppressed mice for an unknown number of years. Mouse propagation of *C. parvum* was approved by and performed in accordance with Tufts University Institutional Animal Care and Use Committee.

**Table 1:** Mapping statistics for 37 RNA-Seq datasets.

ID*	Stage	Sample	Sequences analyzed	Host**		Cryptosporidium parvum	
				Mapped sequences	Mapped %	Mapped sequences	Mapped %
15	Extracellular	Oocysts	7,437,619	9,636	0.13	6,249,367	84.02
16M	Extracellular	Oocysts	12,000,000	5,449	0.05	10,503,020	87.53
1	Extracellular	Sporozoites_2h	11,525,085	5,535	0.05	10,648,804	92.40
2	Extracellular	Sporozoites_2h	9,550,531	11,742	0.12	8,874,905	92.93
3	Extracellular	Sporozoites_2h	8,781,223	5,287	0.06	8,332,943	94.90
4	Extracellular	Sporozoites_2h	9,791,953	7,717	0.08	9,081,237	92.74
5	Extracellular	Sporozoites_2h	8,925,764	4,498	0.05	8,571,776	96.03
6	Extracellular	Sporozoites_2h	9,842,080	8,410	0.09	9,023,619	91.68
7	Extracellular	Sporozoites_0h	10,346,897	2,939	0.03	9,986,685	96.52
8	Extracellular	Sporozoites_0h	7,051,754	4,420	0.06	6,675,995	94.67
9	Extracellular	Sporozoites_2h	8,665,564	3,466	0.04	7,969,618	91.97
10	Extracellular	Sporozoites_2h	9,217,265	10,130	0.11	8,489,030	92.10
11	Extracellular	Sporozoites_0h	10,534,463	5,391	0.05	10,049,403	95.40
12	Extracellular	Sporozoites_0h	9,880,483	7,078	0.07	9,417,974	95.32
13	Extracellular	Sporozoites_2h	12,067,102	5,770	0.05	11,115,287	92.11
14	Extracellular	Sporozoites_2h	8,314,644	11,277	0.14	7,974,108	95.90
1H	Extracellular	Sporozoites_0h	3,000,000	4,074	0.14	769,974	25.70
2H	Extracellular	Sporozoites_0h	3,000,000	3,696	0.12	862,410	28.70
23	Intracellular	infected_MDBK_2h	17,252,177	15,680,504	90.89	68,676	0.39
24	Intracellular	infected_MDBK_2h	19,435,693	17,610,681	90.61	28,243	0.12
25	Intracellular	infected_MDBK_2h	19,063,096	17,286,415	90.68	81,325	0.43
26	Intracellular	infected_MDBK_2h	19,998,766	18,152,880	90.77	43,074	0.22
28	Intracellular	infected_MDBK_24h	20,257,992	17,632,556	87.04	64,235	0.32
29	Intracellular	infected_MDBK_24h	21,596,479	19,417,394	89.91	77,453	0.36
30	Intracellular	infected_MDBK_24h	18,271,010	16,109,550	88.17	69,870	0.38
31	Intracellular	infected_MDBK_24h	17,616,476	15,074,419	85.57	76,016	0.43
1M	Intracellular	infected_IPEC_24h	7,000,000	5,253,821	75.10	136,862	1.96
2M	Intracellular	infected_IPEC_24h	47,904,752	36,263,897	75.70	851,774	1.78
3M	Intracellular	infected_IPEC_24h	7,000,000	5,245,701	74.93	114,070	1.63
4M	Intracellular	infected_IPEC_24h	7,000,000	5,276,146	75.37	115,771	1.65
38	Intracellular	infected_MDBK_48h	16,834,760	15,040,175	89.34	272,638	1.62
39	Intracellular	infected_MDBK_48h	16,504,049	14,734,815	89.28	209,349	1.27
40	Intracellular	infected_MDBK_48h	15,947,732	14,199,861	89.04	191,336	1.20
41	Intracellular	infected_MDBK_48h	18,517,372	16,552,679	89.39	202,993	1.10
42	Intracellular	infected_MDBK_48h	20,299,009	16,630,978	81.93	348,552	1.72
18	Control	uninfected_MDBK	7,000,000	6,258,700	89.41	17,940	0.26
19M	Control	uninfected IPEC-J2	7,000,000	5,628,000	80.40	13,082	0.19

*Sequences of sample IDs designated with “M” are deposited under accession number PRJEB17685 (Mirhashemi *et al*., 2018); sequences of samples indicated with “H” are from (Lippuner *et al*., 2018) (NCBI Sequence Read Archive run # SRR3137248 and SRR3137593). The remaining sequences are deposited under accession numbers PRJEB25665 and PRJEB28268.

**Host: Sporozoites and oocyst sample 15 were mapped to *Mus musculus*; oocyst sample 16 M, and samples from MDBK cells were mapped to the genome of *Bos taurus*; sequence reads from IPEC-J2 were mapped to the *Sus scrofa* genome.

Oocysts were purified from mouse or calf feces on step gradients of 15%/25% (w/v) Nycodenz (Alere Technologies, Oslo, Norway) as described^13^. To obtain sporozoites, purified oocysts were surface-sterilized with 10% commercial bleach (0.5% sodium hypochlorite) for 10 min on ice. Bleach was removed by precipitating the oocysts and resuspending them in sterile PBS (Fig. S2). Magnetic beads (Dynabeads Protein G, Invitrogen) were reacted with monoclonal antibody 5F10 (a gift from Abhineet Sheoran, Cummings School of Veterinary Medicine). Bead-antibody conjugate was mixed with the oocyst suspension and the suspension incubated in 0.75% taurocholic acid to induce oocyst excystation. Unexcysted oocysts and empty oocyst walls were separated from the sporozoites by capturing the beads and attached oocysts and oocyst walls in a magnetic stand. The unbound sporozoites left in suspension were recovered by centrifugation.

RNA was extracted from sporozoites either immediately after purification, designated in Table 1 as “sporozoite_0h” or after a 2-h incubation at 37 °C/5% CO_2_ in RPMI-1640 medium supplemented with 10% FBS and 10 μM N-acetylgalactosamine (GalNAc) (Table 1; sporozoites_2h). This treatment was intended to assess whether the effect GalNAc has on sporozoite morphology^14^ is reflected in the transcriptome.

Animal experiments were performed in accordance with protocols approved by the Tufts University Institutional Animal Care and Use Committee.

### Infection of cell monolayers

Monolayers of MDBK cells (Madin Darby Bovine Kidney, ATCC CCL-22^15^) were infected with *C. parvum* as described previously^16–18^. Monolayers of pig epithelial cells (IPEC-J2)^19^ were grown and infected according to previously described procedures^10^. MDBK cells were grown to near-confluence in 25 cm^2^ flasks in Dulbecco’s Modification of Eagle’s Medium (DMEM, Life Technologies) supplemented with 10% fetal bovine serum, 1% L-glutamine, 100 units/ml penicillin and 100 μg/ml streptomycin. Confluent or near-confluent cell monolayers were infected with surface-sterilized *C. parvum* oocysts at a dose of approximately 1 oocyst/cell, equivalent to 1.3 × 10^5^ oocysts per cm^2^ monolayer or a corresponding dose of 4 sporozoites per cell, taking into consideration that each oocyst contains 4 sporozoites. Following infection, cultures were incubated at 37 °C/5% CO_2_. Infected cultures were incubated for 2 h, 24 h or 48 h. The experiments were replicated as follows: 4 cultures of MDBK cells incubated for 2 h; 4 cultures of MDBK cells incubated for 24 h, and 5 cultures of MDBK cells incubated for 48 h. RNA-Seq data from 4 infected IPEC-J2 cultures harvested 24 h post-infection^10^ were also included. Following incubation for 2 h, 24 h or 48 h, monolayers were washed twice with PBS to remove extracellular sporozoites and oocysts. A sample of cells was processed for immunofluorescence^20^ to confirm the infection. Intracellular development stages were visualized with monoclonal antibody 2E5^18^. For RNA extraction, cells were released from the substrate with a brief incubation in 2 ml Accutase (Millipore Sigma, Burlington, Massachusetts) per 25-cm^2^ surface.

### Molecular biology

RNA was extracted from cells in a Qiacube instrument using an RNeasy kit (Qiagen, Hilden, Germany). RNA from 4 uninfected monolayers of MDBK cells and from uninfected IPEC-J2 cells was extracted in parallel. Prior to extraction, the cells were homogenized in a Minibeadbeater in the presence of 0.5 mm diameter zirconia beads. Extraction of RNA from sporozoites and oocysts was initiated with the same disruption procedure. Guanidine thiocyanate buffer RLT supplemented with 1% β-mercaptoethanol was added to the lysate and the samples transferred to QIAshredder spin columns. RNA was extracted from the filtrate following Qiagen’s RNeasy procedure.

The quality of the RNA was initially assessed by determining the RNA Integrity Number (RIN) using an Agilent Bioanalyzer 2100. An Illumina TruSeq Stranded RNA library kit was used to make strand-specific cDNA libraries from polyA selected RNA from a total of 33 oocyst, sporozoite and infected cell samples plus 4 samples each of uninfected MDBK and IPEC-J2 cells. cDNA libraries were subjected to cluster generation and single-end 100-nucleotide sequencing on an Illumina Hi-Seq 2500 at the Tufts Genomics core facility (tucf.org).

### Bioinformatics and statistical analysis

The *C. parvum* IOWA reference genome and annotation file were downloaded from *Cryptosporidium* genomics resource cryptodb.org^21^, release 34, in FASTA and GFF3 format, respectively. The pig (*Sus scrofa*) references genome (susScr3) and annotation file were downloaded from support.illumina.com/sequencing/sequencing_software/igenome.html. The *Bos taurus* bosTau8 and *Mus musculus* mm10 reference genomes provided in the public galaxy instance at usegalaxy.org were used as reference genomes for samples recovered from calf and mouse feces, respectively. To increase the diversity of the samples included in the analysis, RNA-Seq data from two samples of excysted oocysts of *C. parvum* isolate IPZ:CH-Crypto_K6769^9^ were downloaded from NCBI’s sequence read archive (run # SRR3137248 and SRR3137593). The 75-nt reads were downloaded and randomly subsampled to 3 × 10^6^ reads using program sub.sample in *mothur*^22^. Sequence reads in FASTQ format were aligned to the corresponding host genome and to the *C. parvum* genome using program *subread-align*^23^. Alternatively, sequences were mapped in Galaxy^24^ using HiSat2^25^. Genome-wide values of Fragments Per Kilobase of transcript per Million mapped reads (FPKM) were computed using Cufflinks^26^ or StringTie^27^ in Galaxy.

Global differences between FPKM profiles were visualized using Principal Component Analysis (PCA) as implemented in CANOCO^28^. PCA was applied to a matrix of 35 samples x 3888 *C. parvum* genes, where each fields of the table contained the FPKM for the corresponding sample and gene. Values were set to 0 if no sequence mapped to a gene. For certain analyses, FPKM values were normalized by gene by converting them to z scores, i.e., subtracting the mean and dividing by the standard deviation. To test for an association between life cycle stage and gene expression, the FPKM data were analyzed using Redundancy Analysis (RDA)^28^. In this analysis, genes were considered dependent variables, whereas the developmental stage (extracellular or intracellular) represented the independent categorical variable. The percent fit (FitE^29^) of FPKMs across 35 samples with life cycle stage was calculated using RDA. Shannon diversity was calculated in excel as -Σ p_i_ * ln(p_i_), where p_i_ is the proportion of gene *i* FPKM and the sum is over all genes^30^. Genes differentially expressed in different developmental stages were identified with Linear Discriminant Analysis (LDA) as implemented in program LEfSe^31^. The Galaxy interface of the program at http://huttenhower.sph.harvard.edu/galaxy/ was used for this analysis.

For rank-abundance analysis, genes were ranked in order of diminishing FPKM. The most abundant gene was assigned rank 1 and the least abundant the highest rank for a particular sample. Because the number of genes detected in RNA-Seq data varied by sample (range: 805–3706), gene ranks were normalized to 100, such that the least abundant gene was at rank 100. Normalized ranks were then calculated by dividing each rank by the total number of genes detected in a sample and multiplying the ratio by 100.

Groups of orthologous proteins were identified using the OrthoMCL database. The database is part of the Eukaryotic Pathogens bioinformatics resource center^32^.

## Results

### Gene expression in oocyst and intracellular stages

Sequence volume and statistics of sequences mapping to the *C. parvum* and to the respective host genome for 37 samples is shown in Table 1. As quality control, oocyst and sporozoite sequence reads were also mapped to the genome of the respective host species (mouse, pig or cow). Similarly, sequence reads from uninfected IPEC-J2 and MDBK cells were mapped to the *C. parvum* genome. As expected, the proportion of mapped reads in these controls was low, ranging from 0.03% to 0.26%.

**Figure 1:**
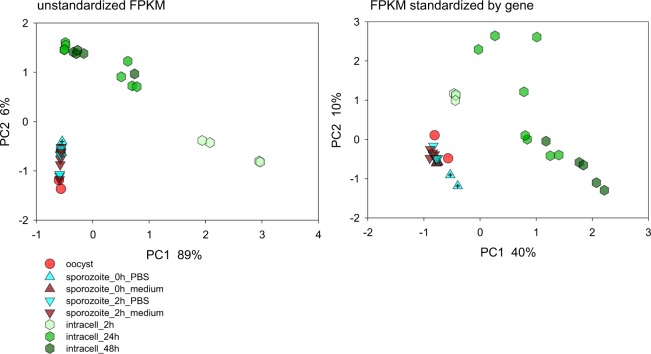
Principal Component Analysis of 35 *Cryptosporidium parvum* transcriptomes. The analysis is based on raw FPKM values for 3885 annotated *C. parvum* genes (left) and FPKM values normalized by gene (right). Included in the analysis are two oocyst samples (red circles), 16 sporozoite samples incubated in medium (brown) or PBS (turquoise) for 0 (triangle up) or 2 h (triangle down) as indicated in the key. Infected cells (hexagons) were analyzed at 2 h, 24 h and 48 h post-infection, as indicated by light, medium and dark green symbols, respectively. Crossed triangles indicate samples 1 H and 2 H (Table 1).

PCA was used to visually compare oocyst, sporozoite and intracellular transcriptome (Fig. 1). The intracellular transcriptome was analyzed at 2 h, 24 h and 48 h. The time points were chosen based on the ability of cultured cell monolayers to support growth of *C. parvum*. PCA was applied to raw FPKM values (Table S1) and to FPKM values normalized by gene. The analysis was duplicated because of the large difference in FPKM values between highly expressed genes and genes expressed at low level; in the analysis based on the raw data, genes with high FPKM are most influential, whereas FPKM normalization by gene gives each gene equal weight. Consistent with differential gene expression during the parasite’s life cycle, PCA clearly discriminated between extracellular parasite stages and intracellular stages, regardless whether FPKM data were normalized or not. The effect of life cycle stage is highly significant based on a randomization of samples with respect to life cycle stages in RDA (pseudo-F = 17.6, p = 0.001). Also apparent in the PCA is the relative similarity between oocyst and sporozoite transcriptome, when compared to the dissimilarity between intracellular and extracellular stages. Incubation of sporozoites post-excystation for 2 h had no apparent effect on the transcriptome profile, regardless whether sporozoites were incubated in PBS or in supplemented culture medium. Significantly, compared to the distance between extracellular and intracellular transcriptome, imported sporozoite RNA-Seq data (Table 1, samples 1 H, 2 H^9^) were similar to those obtained in this study. Whereas extracellular stages form a relatively compact cluster, FPKM profiles of intracellular stages were found to be more heterogeneous. The heterogeneity among intracellular transcriptomes is consistent with the fact that intracellular development was allowed to progress for 2 h, 24 h or 48 h. The distance between intracellular transcriptomes likely reflects differential gene expression during intracellular development, as implied by the clustering of the 2-h samples. This observation is consistent with previously reported differential gene regulation during merogony^33^.

We examined whether oocyst excystation is associated with transcriptome remodeling. To visualize differences between oocyst and sporozoite transcriptomes, PCA was applied to the 18 extracellular samples (2 oocyst and 16 sporozoite samples). Excluding the intracellular samples led to a “decompression” of the 18 extracellular samples on PCA plots, enabling a better visualization of sporozoite-oocysts transcriptome differences (Fig. S3). This analysis revealed that the 16 sporozoite samples generated in this laboratory are similar, regardless of the period and condition in which the sporozoites were incubated post-excystation. The two imported sporozoite transcriptomes generated by Lippuner *et al*. were more distinct, as were the two oocyst transcriptomes. A permutation test showed that in spite of the relatively large difference between the two oocyst samples, sporozoite and oocyst transcriptomes are significantly different (pseudo-F = 3.5; p = 0.02). Together with the PCA of the entire dataset, this analysis indicates that, although extra- and intracellular transcriptomes are clearly distinct, the process of excystation has a detectable impact on the transcriptome. The possible influence of different laboratory procedures used to generate Lippuner’s and our RNA-Seq data on FPKM values revealed in the PCA is discussed below.

**Figure 2:**
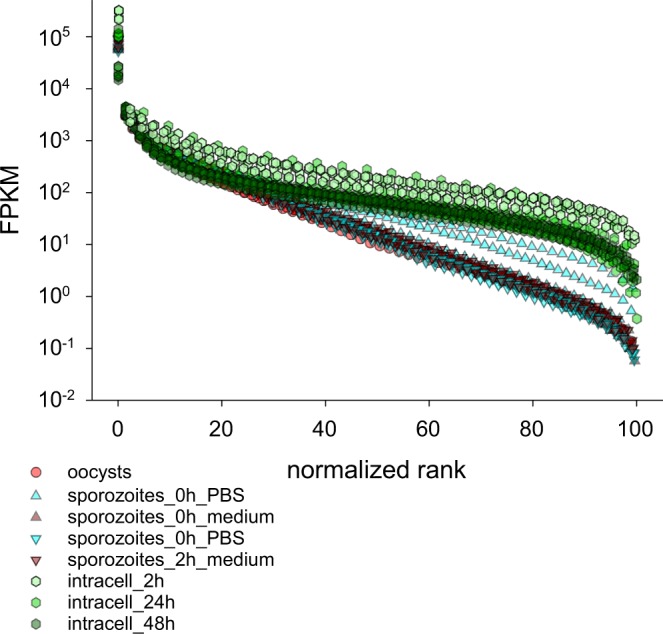
Comparison of normalized rank-abundance plots for 35 C. parvum transcriptomes. Intracellular transcriptomes are more even than transcriptomes from oocysts and sporozoites.

**Figure 3:**
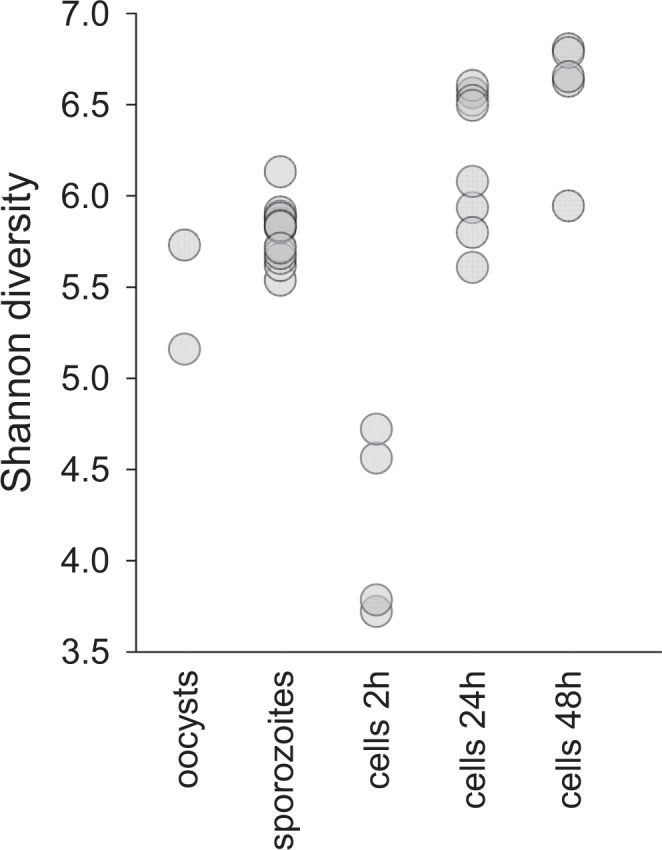
Shannon diversity of 35 C. parvum transcriptomes by life cycle stage. FPKM diversity peaks at 48 h post-infection.

Rank abundance plots were used to visualize differences in the diversity (evenness) of the transcriptome across different stages. This analysis showed that gene expression in intracellular parasites was more even (Fig. 2). As in PCA, oocyst and sporozoite transcriptomes were very similar with respect to evenness. Transcriptome diversity was also estimated using the Shannon diversity index. Based on this metric, the intracellular *C. parvum* transcriptome at 2 h post-infection was the least diverse (mean = 4.19; SD = 0.52, n = 4) (Fig. 3). In contrast, the most diverse *C. parvum* transcriptome was found in intracellular parasites 48 h post-infection (mean = 6.56; SD = 0.35; n = 5). A Kruskal-Wallis ANOVA on Ranks showed that transcriptome diversity varies significantly among life cycle stages (H = 22.86; 4 d.f.; p < 0.001). Because the dataset we analyzed originates from infected cells and from extracellular stages, the number of sequences mapping to the genome of *C. parvum*, as expected, varies widely (Table 1). Between intracellular sample #24 with 28,243 of 19,435,693 (0.12%) mapped sequences and sporozoite sample #13 with 11,115,287 of 12,067,102 mapped sequences (92.11%), there is an almost 400-fold difference in the number of sequences mapping to the *C. parvum* genome. Because the Shannon diversity index is sensitive to sample richness and to population size (in our case the number of mapped sequence reads per sample), we assessed to what extent RNA-Seq Shannon diversity was impacted by sample size. We found little evidence that in our dataset these two variables are correlated (Fig. S4); a linear model fitted to these data shows that sequence abundance only explains 3% of diversity. This observation indicates that the low transcriptome diversity observed in intracellular transcriptomes 2 h post-infection (Fig. 3) is unlikely to be the result of the smaller number of mapped sequences.

In addition to qualitative properties of the intracellular transcriptome, we also examined the proportion of parasite mRNA in the infected cell transcriptome. As expected from the fact that the parasite multiplies during merogony, as *C. parvum* intracellular development progresses, the proportion of parasite transcripts increases (Fig. S5). Consistent with our previous analysis^10^, the parasites transcriptome in cell monolayers does not exceed 2% of the combined host cell – *C. parvum* transcriptome.

### Functional properties of the extra- and intracellular transcriptome

Oocysts and sporozoites are specialized forms of the parasite which have evolved to ensure survival in the environment, delivery of sporozoites to a specific location of the gastro-intestinal (GI) tract^34^ and invasion of the host cell. We tested whether mRNA encoding specialized functions not commonly found among eukaryotes are important constituents of the extracellular parasite transcriptome by tabulating the number of orthologs of the 50 genes most highly expressed in extracellular and intracellular parasite stages, respectively (Tables S2, S3). The number of orthologous genes in eukaryotic pathogens was not significantly different between extracellular and intracellular transcriptome (extracellular, mean = 11.9 orthologs; intracellular mean = 12.2 orthologs: Mann-Whitney Rank Sum Test, p = 0.230). In contrast, the number of orthologs in the 150 genomes found in the OrthoMCL database^35^ was almost double for the genes highly transcribed in the host cell (mean = 133.5) as compared to those highly transcribed outside the host cell (mean = 69.7; Mann-Whitney, p < 0.001). This analysis indicates that, in contrast to intracellular developmental stages, oocysts and sporozoites preferentially express genes encoding specialized functions which have few orthologs outside of related protozoa.

**Figure 4:**
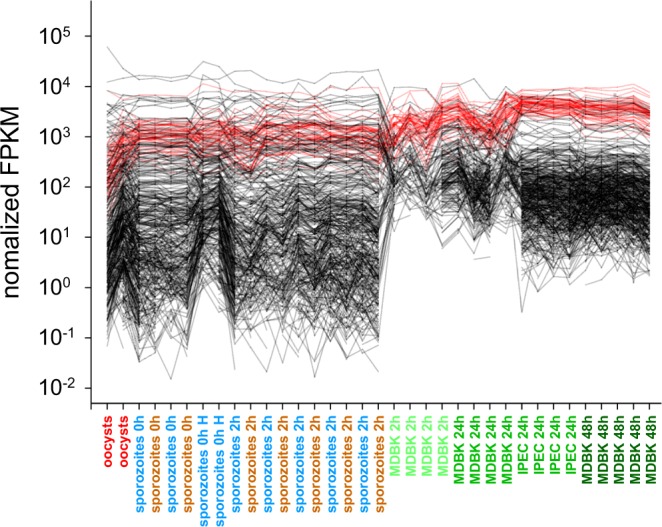
Upregulation of genes encoding ribosomal biosynthesis functions following host cell invasion. Normalized FPKM values of 55 genes encoding ribosomal proteins (red) and 455 randomly chosen genes encoding other functions (black) reveal an upregulation of ribosomal protein expression in relation to other functions. Life cycle stages are ordered on the x axis first in temporal order and second by experiment. The position of replicate samples within each group is arbitrary. The samples are color-coded as in Figs 1 and 2.

We assessed the impact of the transition of non-dividing extracellular life cycle stages to intracellular multiplying stage on functional properties of the parasite transcriptome. Given the importance of ribosomes in the metabolism of dividing cells, the first analysis focused on ribosomal proteins. According to the annotation of the *C. parvum* IOWA genome found in CryptoDB.org, a total of 55 genes encode ribosomal proteins. Functional enrichment analysis of a smaller number of RNA-Seq datasets from intracellular samples^10^, (samples 1 M–4 M, Table 1) had already revealed the abundance of mRNA encoding such proteins 24 h post-infection. The analysis of the expanded dataset (n = 35) confirms this observation (Fig. 4).

**Figure 5:**
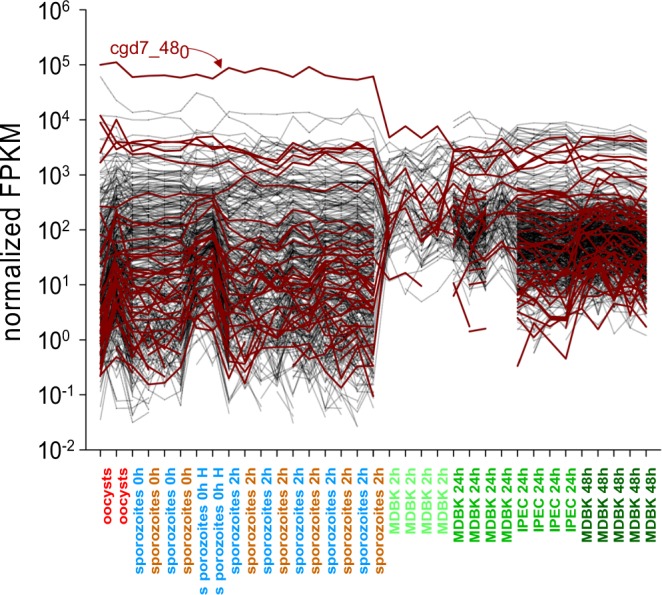
Expression of oxidoreductase functions in oocysts, sporozoites and intracellular developmental stages. Normalized FPKM values of 40 genes encoding oxidoreductase functions (brown) and 460 randomly chosen genes encoding unrelated functions (black) reveal high level of LDH (cgd7_480) transcript in extracellular stages. Life cycle stages are ordered on the x axis in temporal order, then by experiment. The position of samples within each group is arbitrary.

The high level of mRNA transcribed from the *C. parvum* lactate dehydrogenase (LDH) gene cgd7_480, also noticed by Zhang *et al*.^33^, was apparent in our dataset. Particularly striking is the fact that LDH mRNA is by far the most abundant transcript in extracellular stages. To assess whether other genes encoding oxidoreductase functions are similarly regulated, we visualized the FPKM values of 40 *C. parvum* genes with the term “oxidoreductase” in their annotation across the entire RNA-Seq dataset comprising 35 transcriptomes (Fig. 5). The graph shows that other genes in this functional category were also highly expressed in oocysts and sporozoites, but none displayed such and extreme difference between extracellular and intracellular expression as LDH. Using LDA, we also examined the correlation between FPKM and life cycle stage for 21 mRNA transcripts, including LDH, encoding enzymes in the glycolysis pathway (Fig. S6). Unexpectedly, LDH mRNA was clearly unique in the extent of differential regulation among the 21 genes in this metabolic pathway. For instance phosphoglycerate mutase, which is within two metabolic steps of LDH, shows no differential expression in relation to life cycle stages we examined. To ensure that RDA detects co-expression, we ran the same analysis with 7 *C. parvum* genes encoding *Cryptosporidium* oocyst wall proteins and found those to be tightly co-regulated (Fig. S6).

### Differential gene expression

To identify genes that are most differentially expressed between extracellular and intracellular life cycle stages, genes were ranked according to the ratio of mean extracellular FPKM/mean intracellular FPKM. Log_2_ transformed ratios ranged from 5.1 to −16.6. An analysis of enriched biological functions was performed for the 50 genes most overexpressed extracellularly and 50 genes most overexpressed inside the host cell. Gene ontology (GO) enrichment analysis of the former group found no enriched biological function. In contrast, in the 50 genes most overexpressed in the host cell, 10 biological processes were enriched; tetrahydrofolate interconversion (GO:0035999), glycine biosynthetic process from serine (GO:0019264), glycine biosynthetic process (GO:0006545), neurotransmitter biosynthetic process (GO:0042136), L-serine metabolic process (GO:0006563), regulation of neurotransmitter levels (GO:0001505), neurotransmitter metabolic process (GO:0042133), glycine metabolic process (GO:0006544), serine family amino acid biosynthetic process (GO:0009070) and serine family amino acid metabolic process (GO:0009069), (FDR < 0.03). Consistent with the orthology analysis described above suggesting more specialized functions in extracellular stages, there were slightly more uncharacterized genes among the 50 genes most overexpressed in extracellular stages as compared to the 50 genes most overexpressed intracellularly (42% vs 30%). The association between life cycle stage and proportion of uncharacterized genes was, however, statistically not significant (Chi-square 1.6, p = 0.21).

LDA^31^ was used to identify genes that significantly differ in relative transcript abundance between extracellular and intracellular samples. The analysis identified 641 genes with an LDA score >2; 373 of these were overexpressed in oocyst/sporozoites and 268 in the host cell. GO enrichment analysis found 13 biological processes significantly enriched (FDR ≤ 0.05) in extracellularly overexpressed genes (Table 2), and 28 processes significantly enriched in intracellularly overexpressed genes (Table 3). GO analysis revealed a striking difference between biological processes enriched extra- and intracellularly; in extracellular transcriptomes, transmembrane related processes were particularly frequent (5/13 = 38%) as compared to their occurrence in intracellular transcriptomes (0/28). In contrast, and consistent with the analysis of ribosomal proteins illustrated in Fig. 4, GO terms related to biosynthetic processes were significantly more abundant in intracellular transcriptomes (9/28 = 32%) as compared to extracellular transcriptomes, where this term was not enriched. The association between GO term and life cycle stage was significant by Fisher Exact Test for both processes (transmembrane, p = 0.002; biosynthetic p = 0.038).

**Table 2:** GO term enrichment analysis of 373 genes overexpressed in extracellular development stages of *Cryptosporidium parvum*.

ID	Name	Fold enrichment	FDR
GO:0042254	ribosome biogenesis	6	1.18E-08
GO:0022613	ribonucleoprotein complex biogenesis	5.78	1.364E-08
GO:0044085	cellular component biogenesis	4.08	4.169E-06
GO:0055085	transmembrane transport	3.23	2.858E-05
GO:0016072	rRNA metabolic process	5.44	0.001
GO:0006364	rRNA processing	5.44	0.001
GO:0071840	cellular component organization or biogenesis	2.58	0.002
GO:0099132	ATP hydrol coupled cation transmembr transport	4.08	0.045
GO:0099131	ATP hydrol coupled ion transmembrane transport	4.08	0.045
GO:0015991	ATP hydrolysis coupled proton transport	4.08	0.045
GO:0015988	energy coupled proton transmembr transport, against electrochemical gradient	4.08	0.045
GO:0090662	ATP hydrolysis coupled transmembrane transport	4.08	0.045
GO:0016070	RNA metabolic process	1.79	0.046

**Table 3:** GO term enrichment analysis of 268 genes overexpressed in intracellular development stages of *Cryptosporidium parvum*.

ID	Name	Fold enrichment	FDR
GO:0043043	peptide biosynthetic process	8.29	3.90E-60
GO:0006412	translation	8.29	3.90E-60
GO:0043604	amide biosynthetic process	8.21	8.38E-60
GO:0006518	peptide metabolic process	8.06	6.11E-59
GO:0043603	cellular amide metabolic process	7.98	1.48E-58
GO:1901566	organonitrogen compound biosynthetic process	6.35	8.47E-52
GO:0044271	cellular nitrogen compound biosynthetic process	5.39	4.64E-46
GO:0034645	cellular macromolecule biosynthetic process	4.88	2.72E-39
GO:0009059	macromolecule biosynthetic process	4.85	3.94E-39
GO:1901576	organic substance biosynthetic process	4.1	1.21E-35
GO:0044249	cellular biosynthetic process	4.1	1.21E-35
GO:0009058	biosynthetic process	3.92	6.61E-34
GO:0010467	gene expression	4.26	8.52E-34
GO:0044267	cellular protein metabolic process	3.6	7.27E-33
GO:1901564	organonitrogen compound metabolic process	3.07	6.38E-31
GO:0019538	protein metabolic process	3.2	2.33E-29
GO:0034641	cellular nitrogen compound metabolic process	2.98	3.47E-24
GO:0044260	cellular macromolecule metabolic process	2.67	4.78E-23
GO:0006807	nitrogen compound metabolic process	2.1	1.79E-17
GO:0009987	cellular process	1.92	2.61E-17
GO:0044237	cellular metabolic process	2.09	3.92E-17
GO:0044238	primary metabolic process	1.99	3.68E-16
GO:0071704	organic substance metabolic process	1.96	6.74E-16
GO:0043170	macromolecule metabolic process	2.11	7.09E-16
GO:0008152	metabolic process	1.82	7.34E-15
GO:0008150	biological process	1.45	1.68E-10
GO:0006414	translational elongation	9.99	2.75E-05
GO:0006457	protein folding	3	0.022

## Discussion

To advance our understanding of gene regulation in the *Cryptosporidium* life cycle, here we focused on analyzing changes in the transcriptome associated with the transition from non-dividing extracellular stages to intracellular multiplicative forms. In contrast to the differentiation of gametes, meiosis and formation of oocysts, oocyst excystation can readily be studied *in vitro*, as can the initial phase of merogony in the host cell. Infecting cell monolayers with oocysts or sporozoites generates a reasonably synchronized infection^3^, facilitating the identification of differential gene expression over time. By including samples from three different studies, we ensure that the results are generally valid, i.e., are not restricted to a specific cell line or *C. parvum* isolate. We found that transcriptomes of extracellular developmental stages cluster together, regardless of isolate and sequencing strategy. Similarly, parasite transcriptomes 24 h post-infection originating from two different cell lines also were found to cluster (Fig. 1), although not as tightly as sporozoite and oocyst transcriptomes. These results likely reflect, in part, the impact of different laboratory procedures used to generate RNA-Seq data. The position of the sporozoite datapoints from Lippuner’s study in Fig. S3, relative to the datapoints from the present study, illustrates the magnitude of this effect. Lippuner and co-workers used a shorter excystation procedure (20 min vs. 60 min in our laboratory), a difference that may have contributed to their sporozoite transcriptome profiles being slightly different from ours. Validating the approach of incorporating different datasets, the noise introduced by the experimental variables is small compared to the difference between the transcriptome in different life cycle stages.

The global transcriptome analysis (Fig. 1) shows that oocysts and sporozoites express similar transcriptomes. This does not necessarily exclude differential gene expression during and following excystation. The small number of oocyst samples sequenced in this project limits our ability to investigate the transcriptional basis of excystation. In addition, differences between oocyst transcriptomes apparent in Fig. S1 may have been introduced by differences in oocyst age and storage conditions. Indeed, using quantitative PCR, we found that oocyst age impacts the abundance of selected transcripts^36^. A larger number of oocyst transcriptomes will need to be sequenced to dissect the effect of these experimental variables. The same reasoning applies to the effect of the cell line on *C. parvum* gene expression. Although our analyses include data from infected bovine and porcine cells, it is unknown to what extent the cell line has impacted *C. parvum* gene expression, since these samples originated from different experiments. Other variables, such as the degree of confluence of the cell monolayer or the number of infectious oocysts per cell would have to be considered. These analyses require a larger number of samples than analyzed here.

A general limitation of existing methods to culture *Cryptosporidium* parasites is that the infection is patchy and many host cells remain uninfected. The proportion of infected cells is difficult to measure and to control, precluding an estimation of the true proportion of *C. parvum* transcripts in the in the infected cell transcriptome. This limitation is reflected in the varying percentage of intracellular sequence reads mapping to the *C. parvum* genome (Table 1).

Only a small number of published studies have focused on *Cryptosporidium* transcriptomics. To our knowledge, Lippuner’s study^9^ is the only publication based on RNA-Seq to characterize the transcriptome of different *C. parvum* life cycle stages. In light of the inclusion of data from this study into our analyses, we examined the extent to which our data correlated with those previously reported^9^. Since both studies sequenced sporozoite transcriptomes and the intracellular transcriptome 48 h PI, these stages were included in the comparison. This analysis returned a correlation coefficient (r^2^) of 0.72. This result has to be evaluated in light of the differences between studies, like laboratory procedures, biological material (cell lines and *C. parvum* isolate) and sequencing strategy. The relatively high correlation between data from independent studies is encouraging, demonstrating that RNA-Seq is a robust approach to analyzing the complexity of the *C. parvum* transcriptome, even in the presence of an overwhelming excess of host RNA. This result also argues in favor of combining datasets from different studies as a way of ascertaining the general validity of the results.

The present study confirmed the abundance of LDH mRNA in oocysts previously reported^33^, and shows that this feature is characteristic of all extracellular stages studied here. What is particularly intriguing about this observation is that among 21 genes encoding enzymes in the glycolytic pathway only LDH mRNA is expressed at such high level. The functional significance of this observation remains to be elucidated.

## Conclusions

RNA-Seq analysis of the *C. parvum* trophozoite/meront transcriptome and of extracellular stages (sporozoites and oocysts) has revealed significant differences in gene expression, both in terms of diversity and function. Whereas in the intracellular transcriptome, functions related to ribosomes and protein synthesis are highly enriched, the oocyst transcriptome does not have a clear functional signature. Genes highly expressed in oocysts appear to fulfill more specialized functions as indicated by a smaller number of orthologs in other eukaryotic genomes.

## Supplementary information

Supplementary Information

Dataset 1

Dataset 2

Dataset 3

## Data Availability

Sequence data were deposited in the European Nucleotide Archive under project accession numbers PRJEB25665 and PRJEB28268. Transcriptome data were also deposited with CryptoDB (www.cryptodb.org).
