# Supplementary material for: The transcriptome of Cryptosporidium oocysts and intracellular stages
Source: Sci Rep. 2019 May 27;9:7856. doi: 10.1038/s41598-019-44289-x (PMC6536522; doi:10.1038/s41598-019-44289-x)
Supplement: Supplementary file 1 — Supplementary Information [file 41598_2019_44289_MOESM1_ESM.pdf]

Supplementary Information

The transcriptome of *Cryptosporidium* oocysts and intracellular stages

Lucas V. S. Matos, John McEvoy, Saul Tzipori, Katia D. S. Bresciani, Giovanni Widmer

Supplemental material

Table S1. FPKM values for 35 samples and 3793 *Cryptosporidium parvum* genes.

Table S2. Ortholog counts of 50 genes most highly expressed in oocysts/sporozoites.

Table S3. Ortholog counts of 50 genes most highly expressed in trophozoites/meronts (intracellular stages).

Fig. S1. Flowchart of experimental procedures.

Fig. S2. Micrograph of a sporozoite preparation.

Fig. S3. PCA of 18 RNA-Seq datasets from *Cryptosporidium parvum* oocysts and sporozoites. Crossed triangles represent transcriptomic data generated by Lippuner et al.

Fig. S4. Lack of correlation between the number of sequences mapping to the *C. parvum* genome and Shannon diversity for 35 samples.

Fig. S5. Percent of RNA-Seq sequence reads mapping to the *C. parvum* genome at three timepoints post-infection.

Fig. S6. Correlation between transcript abundance and life cycle stage.

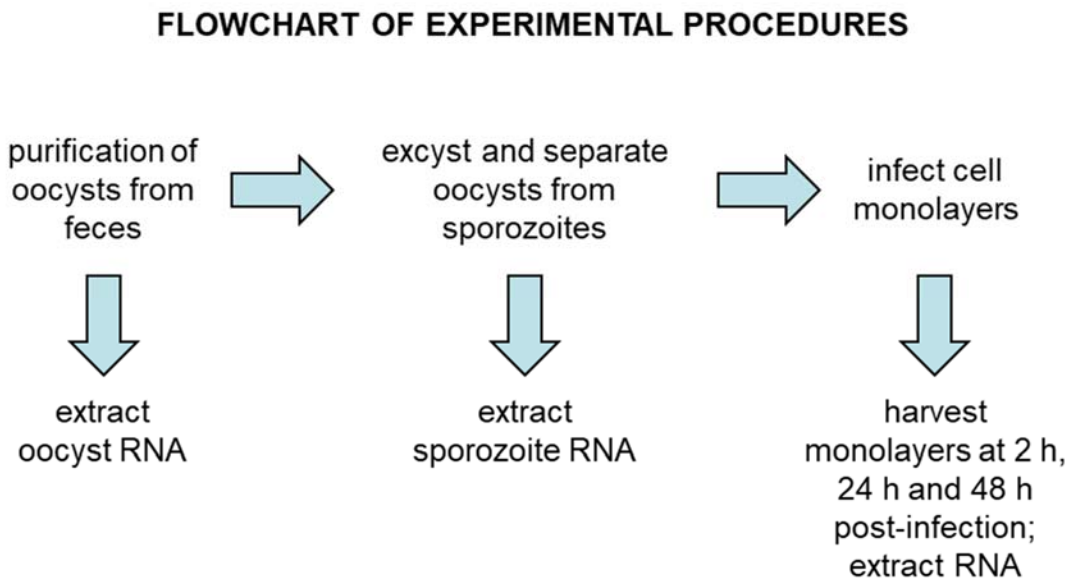

Fig. S1. Experimental procedures.

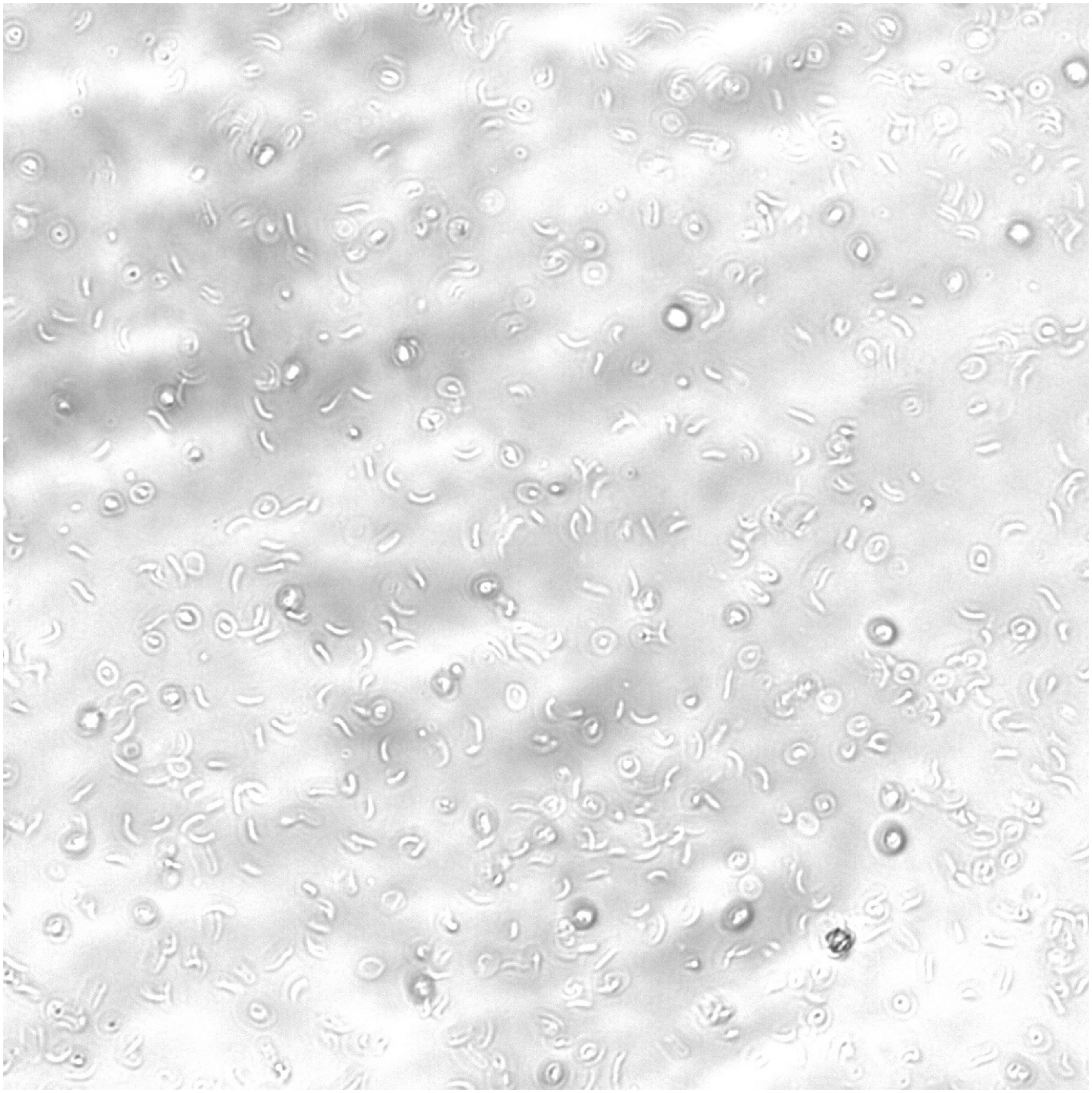

Fig. S2. Phase contrast micrograph of a preparation of sporozoites used in the experiments. Comma-shaped structures are sporozoites, low-contrast spherical structures are empty oocysts and high-contrast spherical structures are unexcysted oocysts which co-purified the sporozoites. Oocysts are approximately 4.5 μm in diameter.

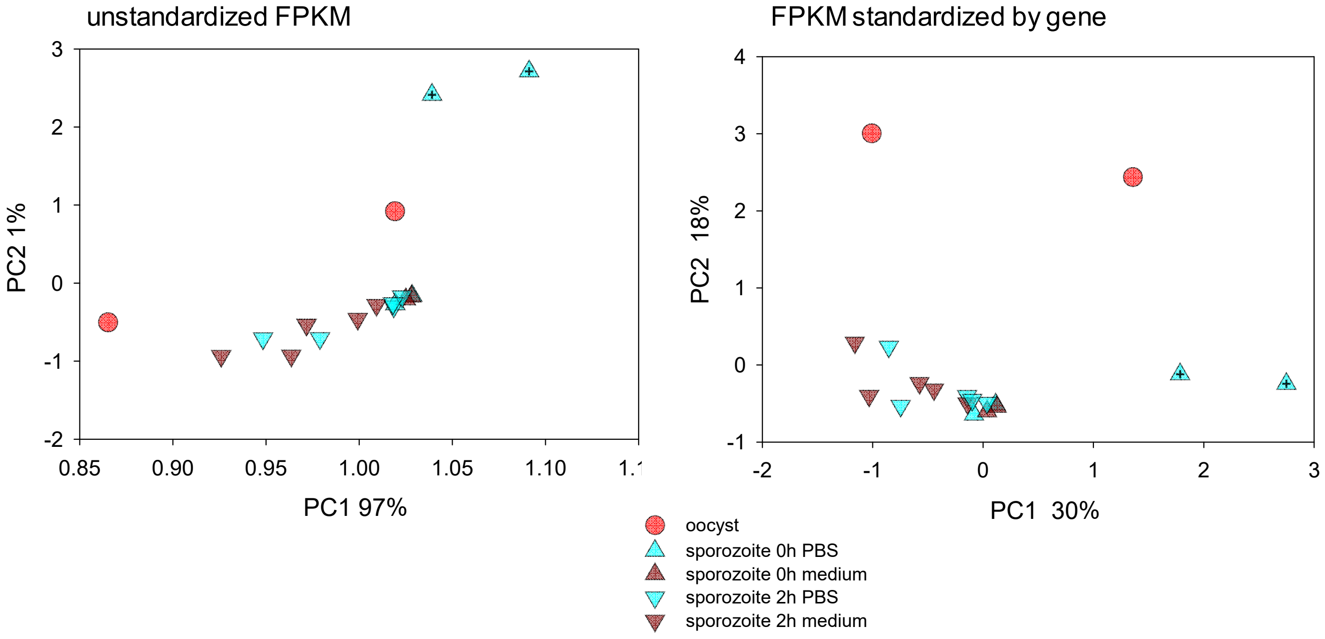

Fig. S3. Principal Component Analysis of 18 RNA-Seq datasets from *Cryptosporidium parvum* oocysts and sporozoites. Crossed triangles represent RNA-Seq data generated by Lippuner et al. The percent of variance explained by each axis is indicated.

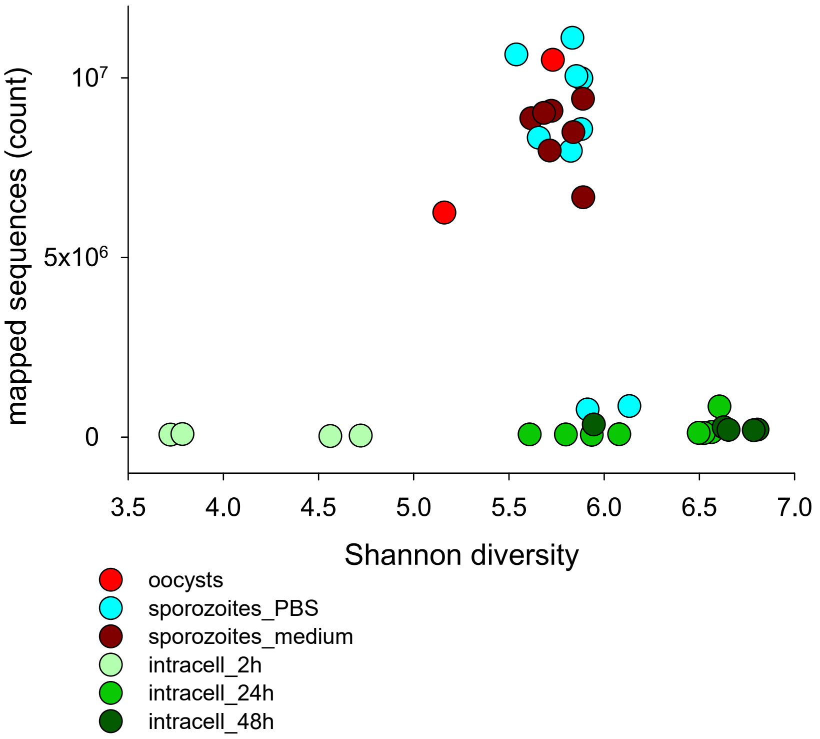

Fig. S4. Lack of correlation between the number of sequences mapping to the *C. parvum* genome and Shannon diversity for 35 samples. Fewer sequences from intracellular transcriptomes map to the genome of *C. parvum* because of the predominance of host transcripts in infected cells.

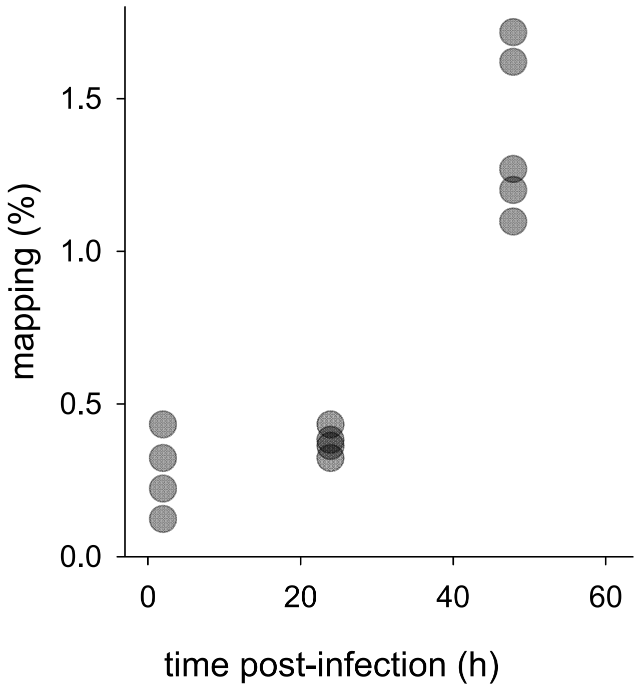

Fig. S5. Percent of RNA-Seq reads mapping to the *C. parvum* genome at three timepoints post-infection. Merogony is associated with an increase in the fraction of *C. parvum* transcripts in the host-parasite transcriptome.

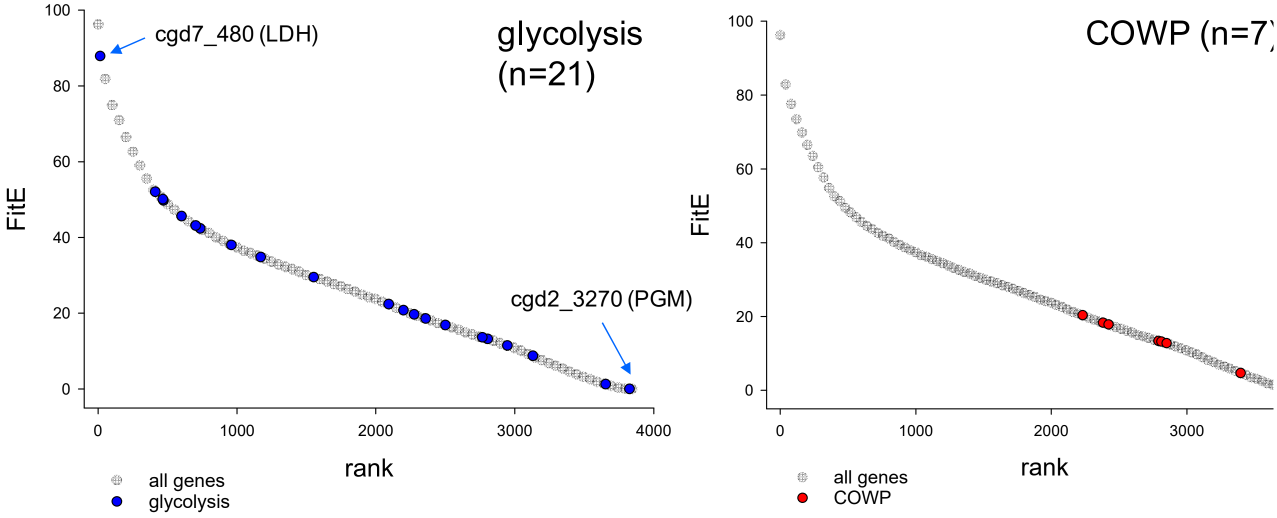

Fig. S6. Correlation between transcript abundance and life cycle stage. FitE values plotted on the y axis represent the percent fit of FPKM for each gene explained by the extracellular stage, where 100% indicates a perfect correlation and 0% no correlation. The entire *C. parvum* genome (3857 genes) was ranked in order of decreasing FitE. The 21 glycolysis genes highlighted in blue in the left panel show a wide range of fit, from 87.9% for cgd7_480 down to 0.03% for cgd2_3270, indicating widely different expression patterns in this pathways. A control analysis with 7 genes encoding *Cryptosporidium* oocyst wall proteins (COWP), expected to be co-expressed during the life cycle, showed a much narrower FitE range, consistent with transcriptional co-regulation. The low COWP FitE values are expected based on the fact that oocyst wall synthesis does not occur in the life cycle stages examined in this study.
